# Clevidipine vs. nicardipine in hypertensive crisis: a systematic review and meta-analysis

**DOI:** 10.1186/s12872-026-06345-z

**Published:** 2026-07-24

**Authors:** Hassaan Abid, Syed Mohamin Abbas Shah, Amina Zia Rashid, Bisma Tasawar, Rimsha Adnan, Sohana Memon, Gaaitri Lohano, Muhammad Jawad, Muhammad Vazaym, Muhammad Junaid

**Affiliations:** 1 Indiana University School of Medicine, Muncie, Indiana United States; 2 King Edward Medical University, Lahore, Punjab Pakistan; 3 Allama Iqbal MedicalCollege, Lahore, Punjab Pakistan; 4 Dow University of Health Sciences (DUHS), Karachi, Pakistan; 5 Liaquat University of Medical & Health Sciences, Jamshoro, Sindh Pakistan; 6 Khyber Medical College Peshawar, Peshawar, Pakistan

**Keywords:** Hypertensive crisis, Hypertensive emergency, Clevidipine, Nicardipine, Calcium channel blocker

## Abstract

**Background:**

Clevidipine and nicardipine are commonly used intravenous calcium channel blockers for the management of hypertensive crises. However, comparative evidence regarding their efficacy and safety remains limited.

**Methods:**

We conducted a systematic review and meta-analysis of randomized controlled trials and observational studies comparing intravenous clevidipine and nicardipine in adults with hypertensive crises. The primary outcome was achievement of target blood pressure reduction. Secondary outcomes included medication volume and blood pressure over-reduction. Random-effects models were used, and certainty of evidence was assessed using the GRADE framework.

**Results:**

Three studies involving 599 patients were included. Clevidipine and nicardipine demonstrated comparable achievement of target blood pressure reduction (OR 0.96, 95% CI 0.48–1.94; I² = 60%; very low-certainty evidence). Clevidipine was associated with lower odds of blood pressure over-reduction (OR 0.44, 95% CI 0.21–0.93; I² = 0%) and lower medication volume (MD − 258.43 mL, 95% CI − 313.38 to − 203.48 mL; I² = 0%).

**Conclusion:**

Clevidipine and nicardipine demonstrated comparable efficacy in achieving target blood pressure reduction in hypertensive crises. Although clevidipine was associated with lower odds of blood pressure over-reduction and lower medication volume, the evidence base was limited and the certainty of evidence for the primary outcome was very low. These findings should be interpreted cautiously and considered hypothesis-generating until confirmed by larger, high-quality randomized controlled trials.

**Supplementary Information:**

The online version contains supplementary material available at https://doi.org/10.1186/s12872-026-06345-z.

## Introduction

Elevated blood pressure remains the leading preventable risk factor for premature death and disability worldwide and is a major contributor to cardiovascular, cerebrovascular, and renal diseases [1]. A hypertensive crisis is characterized by a sudden, marked elevation in blood pressure and is classified as a hypertensive emergency when accompanied by acute target-organ damage or as a hypertensive urgency in its absence [2]. Prompt but controlled blood pressure reduction using titratable intravenous antihypertensive agents is essential to limit ongoing end-organ injury while avoiding excessive hypotension [3, 4].

Among intravenous antihypertensive agents, clevidipine and nicardipine are widely used because of their potent arterial vasodilatory effects and favorable hemodynamic profiles [5]. Clevidipine is an ultra-short-acting intravenous dihydropyridine calcium channel blocker that is rapidly metabolized by blood and tissue esterases, resulting in a very short half-life that enables precise dose titration, predictable blood pressure control, and use independent of renal or hepatic function [6]. In contrast to many other vasodilators, clevidipine primarily acts on the arterial circulation with minimal effects on preload, contributing to its hemodynamic stability [6].

Recent studies have shown that both clevidipine and nicardipine are effective for achieving target blood pressure in hypertensive crises and other acute cardiovascular and neurological conditions [5]. Owing to its unique pharmacokinetic profile, clevidipine has demonstrated rapid achievement and maintenance of target blood pressure with a favorable safety profile [7]. Moreover, contemporary evidence suggests that clevidipine provides blood pressure-lowering efficacy comparable to nicardipine while offering the advantage of rapid onset, rapid offset, and easy titration during continuous infusion [5, 6].

Previous systematic reviews have primarily evaluated the safety and efficacy of clevidipine in specific clinical settings, particularly among neurocritical and cerebrovascular patients, thereby limiting the generalizability of their findings to the broader population with hypertensive crises [8, 9]. In addition, available comparative studies of clevidipine and nicardipine have reported heterogeneous findings regarding blood pressure control and safety outcomes, and recent comparative evidence has not yet been comprehensively synthesized [5, 7]. Therefore, an updated systematic review and meta-analysis is warranted to provide robust evidence on the comparative efficacy and safety of clevidipine versus nicardipine in patients with hypertensive crises. Accordingly, the present study aims to compare the efficacy and safety of clevidipine and nicardipine, with a particular focus on blood pressure control, medication requirements, and safety outcomes.

## Methods

### Study design and protocol registration

This meta-analysis was conducted in accordance with the Cochrane Handbook for Systematic Reviews of Interventions and reported in accordance with the Preferred Reporting Items for Systematic Reviews and Meta-Analyses (PRISMA) statement [10]. The study protocol was registered in PROSPERO (Registration ID: CRD420251166521).

### Data resources and search strategy

A comprehensive literature search was performed using PubMed, Embase, Scopus, and the Cochrane Library from database inception to January 2026. The search strategy included a combination of keywords, Medical Subject Headings (MeSH), and synonyms related to “clevidipine” and “hypertensive crisis,” combined using Boolean operators (AND, OR).

Reference lists of all included studies were manually screened to identify additional eligible publications. Only studies published in English were included. Grey literature, including conference abstracts, dissertations, and unpublished studies, was excluded due to incomplete methodological details and lack of peer-reviewed validation. Duplicate records were removed prior to screening. The detailed search strategies for each database are provided in [Table 1].

**Table 1:** Search string

Database	Search String
Embase	(‘clevidipine’/exp OR clevidipine OR ‘clevidipine butyrate’ OR ‘clevidipine-butyrate’ OR cleviprex OR ‘dihydropyridine derivative’/exp OR ‘calcium channel blocker’/exp) AND (‘hypertensive crisis’/exp OR ‘hypertensive emergency’/exp OR ‘hypertensive urgency’/exp OR ‘hypertensive crisis’ OR ‘hypertensive crises’ OR ‘hypertensive emergency’ OR ‘hypertensive emergencies’ OR ‘hypertensive urgency’ OR ‘hypertensive urgencies’)
Pubmed	(“clevidipine“[Supplementary Concept] OR clevidipine OR “clevidipine butyrate” OR “clevidipine-butyrate” OR Cleviprex OR Dihydropyridines OR “Calcium Channel Blockers“[MeSH]) AND (“Hypertensive Crisis“[MeSH] OR “Crises, Hypertensive” OR “Crisis, Hypertensive” OR “Hypertensive Crises” OR “Hypertensive Emergency” OR “Emergencies, Hypertensive” OR “Emergency, Hypertensive” OR “Hypertensive Emergencies” OR “Hypertensive Urgency” OR “Hypertensive Urgencies” OR “Urgencies, Hypertensive” OR “Urgency, Hypertensive”)
Scopus	TITLE-ABS-KEY (clevidipine OR “clevidipine butyrate” OR “clevidipine-butyrate” OR Cleviprex OR dihydropyridines OR “calcium channel blockers”) AND TITLE-ABS-KEY (“hypertensive crisis” OR “hypertensive crises” OR “hypertensive emergency” OR “hypertensive emergencies” OR “hypertensive urgency” OR “hypertensive urgencies”)
Cochrane	(clevidipine OR “clevidipine butyrate” OR clevidipine butyrate OR Cleviprex OR dihydropyridines OR “calcium channel blockers”) AND (“hypertensive crisis” OR “hypertensive crises” OR “hypertensive emergency” OR “hypertensive emergencies” OR “hypertensive urgency” OR “hypertensive urgencies”)

### Study selection and eligibility criteria

All retrieved records were imported into Rayyan AI for screening. Two reviewers (BT and AR) independently screened titles and abstracts, followed by full-text evaluation of potentially eligible studies. Discrepancies were resolved through discussion, with arbitration by a third reviewer (HA) when necessary. Details of excluded studies are present in Supplemental Table 1.

The eligibility criteria were as follows: (1) adult patients (≥ 18 years) presenting with hypertensive crises; (2) evaluation of intravenous clevidipine vs. intravenous nicardipine; (3) randomized controlled trials or observational studies (cohort or case-control); (4) reporting at least one efficacy or safety outcome related to blood pressure control in hypertensive crisis.

### Data extraction and outcomes

Two authors (BT and AR) independently extracted data using a pre-defined Microsoft Excel spreadsheet. Any conflicts during extraction were resolved by a third author (HA). Data were extracted from study text, tables, and figures, with raw values estimated from percentages where necessary. Extracted information included study characteristics (first author, publication year, study type, and sample size), patient demographics (age and sex), and baseline clinical characteristics when available. Details of the interventions, including drug type, dosing, and administration, were also recorded, along with the duration of follow-up.

The primary outcome was achievement of target blood pressure reduction, defined according to the endpoint reported in each included study. Outcome definitions varied across studies with respect to the blood pressure metric (e.g., systolic blood pressure or mean arterial pressure), target blood pressure reduction, and timing of assessment. These data were extracted as originally reported. Although the operational definitions were not identical, all included studies evaluated early achievement of protocol- or guideline-defined blood pressure reduction following initiation of intravenous antihypertensive therapy and were therefore considered clinically comparable for quantitative synthesis. Secondary outcomes included total medication volume required and the incidence of blood pressure over-reduction or adverse events. Detailed study-specific outcome definitions are provided in [Supplementary Table S1].

### Quality assessment

The risk of bias for randomized controlled trials was assessed using the revised Cochrane Risk of Bias tool (RoB 2) [11]. This tool evaluates bias across five domains: [1] bias arising from the randomization process [2], deviations from intended interventions [3], missing outcome data [4], measurement of outcomes, and [5] selection of reported results. Each trial’s overall risk of bias was categorized as low, some concerns, or high.

The two cohort studies were assessed using the Newcastle–Ottawa Scale (NOS) [12].

Two reviewers (BT and AR) independently performed the assessments, with disagreements resolved through discussion and, if necessary, consultation with a third reviewer (HA).

### Certainty of evidence assessment

The certainty of evidence for each outcome was assessed using the Grading of Recommendations Assessment, Development and Evaluation (GRADE) framework. Because the available evidence included both randomized controlled trials and observational studies, certainty was assessed at the outcome level by considering the overall body of evidence contributing to each pooled estimate. Judgements were made in accordance with published GRADE guidance, taking into account study design, risk of bias, inconsistency, indirectness, imprecision, and publication bias when assessable. Downgrading decisions were applied when concerns were identified in these domains. Upgrading criteria for observational evidence (e.g., large magnitude of effect, dose–response relationship, or residual confounding likely to reduce the observed effect) were also considered but were not met for any outcome [Table 2]. 

**Table 2:** GRADE assessment

Outcome	Sample Size	I²(%)	Effect Estimate(95%CI)	Quality Assessment	Inconsistency	Indirectness	Imprecision	Publication Bias	Overall Quality
Achievement of target blood pressure reduction	599(2Cohorts+1RCT)	60%	0.96[0.48,1.94]	Not serious	Serious	Not serious	Serious	N/A	Very low
Medication Volume	443(1Cohort+1RCT)	0%	-258.43[-313.38,-203.48]	Serious	Not serious	Not serious	Not serious	N/A	Low
BP Over Reduction	409(1Cohort+1RCT)	0%	0.44[0.21,0.93]	Not serious	Not serious	Not serious	Not serious	N/A	Moderate

### Statistical analysis

All analyses were conducted using Review Manager (RevMan) version 5.4.1. For dichotomous outcomes, pooled estimates were calculated as odds ratios (OR) with 95% confidence intervals (CIs) using the Mantel–Haenszel random-effects model. For continuous outcomes, pooled effects were calculated using the inverse-variance random-effects method and reported as mean differences where appropriate. Results were presented using forest plots. A *p*-value < 0.05 was considered statistically significant.

Statistical heterogeneity was assessed using the Chi-square test (*p* < 0.10 indicating heterogeneity) and quantified using the I² statistic. I² values below 50% were considered low to moderate heterogeneity, while values of 50% or above indicated substantial heterogeneity.

To explore potential sources of heterogeneity, subgroup analyses were performed based on study design (randomized controlled trials versus cohort studies) when feasible. A leave-one-out sensitivity analysis was conducted by sequentially removing one study at a time to evaluate the robustness of the pooled estimates.

Randomized controlled trials and observational studies were pooled together using a random-effects model due to anticipated clinical and methodological heterogeneity and the limited number of available studies. Formal assessment of publication bias (e.g., funnel plot asymmetry or Egger’s regression test) was not performed because fewer than 10 studies were included, consistent with recommended methodological guidance.

## Results

### Search results

**Fig. 1:**
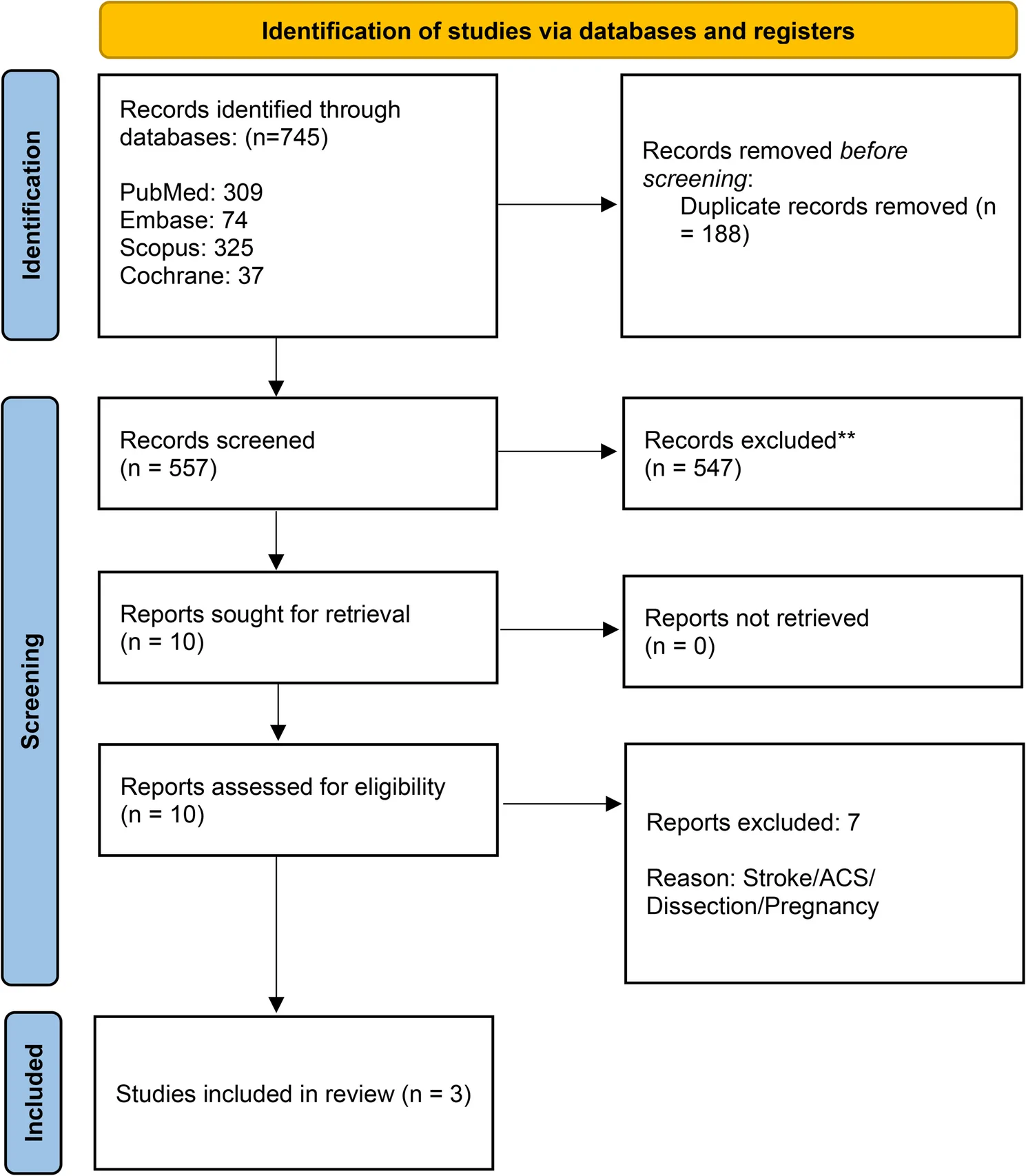
PRISMA Flowchart

The search identified 745 records: 325 from Scopus, 74 from Embase, 309 from PubMed, and 37 from Cochrane. After removing 188 duplicate records, 557 studies remained for the title and abstract screening. Following the screening process, 547 studies were excluded based on title and abstract evaluation. 10 were assessed for eligibility, of which 3 studies [5, 13, 14] were included in the qualitative and quantitative synthesis. The detailed screening process is illustrated in [Fig. 1].

### Study characteristics

A total of 599 patients were included across the analyzed studies, with 291 patients receiving clevidipine and 308 receiving nicardipine. The studies were published between 2024 and 2026 and comprised two cohort studies and one randomized controlled trial. Two studies were conducted in the United States, and one study was conducted in China.

Neither observational study reported the use of propensity score matching; therefore, the pooled observational evidence remains susceptible to residual confounding. The mean age of participants ranged from 51.6 to 64 years across treatment groups. Female representation ranged from 39.7% to 50.5%. Baseline systolic blood pressure was generally markedly elevated, with reported mean values ranging from approximately 179 to 201 mmHg, and the majority of patients presented with hypertensive emergency. A high prevalence of pre-existing hypertension was observed across studies, while histories of atrial fibrillation were less common. Detailed study-level and patient-level baseline characteristics are provided in [Tables 3 and 4].

**Table 3:** Study characteristics

Author, Year	Country	Route of Administration	Type of Study	Intervention	Control	SAMPLE SIZE	
						C	*N*
Johnson 2024 [13]	USA	IV (both arms)	Cohort	Clevidipine (Starting 2.5 mg/h)	Nicardipine IV (Starting 1 mg/h)	79	103
Storey 2024 [5]	USA	IV (both arms)	Cohort	Clevidipine	Nicardipine	82	74
Lv 2026 [14]	China	IV (both arms)	RCT	Clevidipine (Starting 2 mg/h)	Nicardipine IV (Starting 0.5 mg/h)	130	131

*IV* Intravenous, *RCT* Randomized Control Trial

**Table 4:** Baseline characteristics

	Johnson 2024 [13]		Storey 2024 [5]		Lv 2026 [14]	
	C	*N*	C	*N*	C	*N*
Age (yrs) Mean (SD)	60.7 ± (1.8)	58.1 ± (1.6)	59 ± 17	64 ± 18	53.1 ± 12.2	51.6 ± 13.2
Female (%)	35 (44.3%)	52 (50.5%)	60 (46.2%)	52 (39.7%)	60 (46.2%)	52 (39.7%)
Weight (kg) Mean (SD)	83.4 ± (1.6)	80.3 ± (23.3)	88.5 ± 24.1	86.5 ± 29		
Baseline SBP (mmHg) (Mean + SD)	180 ± 42.2	179 ± 37.0	> 180 mm Hg	> 180 mm Hg	201 ± NR	201 ± NR
White (%)	30 (38.0%)	31 (30.1%)	54 (66%)	54 (73%)		
Hypertensive Emergency (%)	100%	100%	82%	80%	100%	100%
PMHx of HTN (%)	80%	82%	84%	80%		
PMHx of Afib (%)	15.20%	16.50%	10%	10%		

*C* Clevidipine, *N* Nicardipine, *NR*  not reported, *SBP* Systolic Blood Pressure, *Afib* Atrial Fibrillation, *HTN* Hypertension

### Risk of bias assessment

Overall, the two cohort studies were judged to be of high quality, whereas the RCT was rated as having some concerns regarding risk of bias. Detailed domain-level assessments are presented in the corresponding ROB 2.0 and NOS figures and tables [Supplementary Figure S1].

### Outcomes

#### Primary outcome

##### Achievement of target BP reduction

**Fig. 2:**
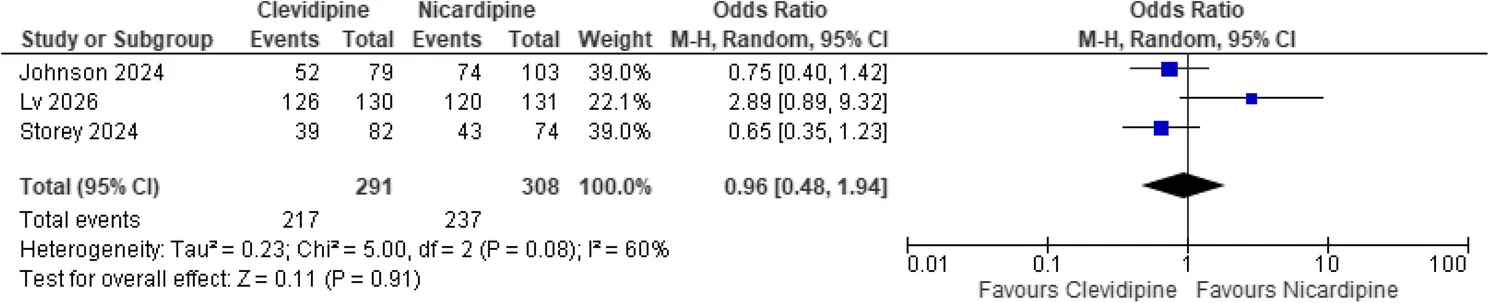
Forest plot of achievement of target blood pressure reduction

Analysis of three studies including 599 patients showed no statistically significant difference between clevidipine and nicardipine in achieving the target blood pressure reduction (OR 0.96, 95% CI 0.48–1.94, *p* = 0.91). Although no statistically significant difference was observed, moderate statistical heterogeneity was present (I² = 60%), likely reflecting differences in study populations, blood pressure targets, outcome definitions, and assessment time points across the included studies. The certainty of the evidence was rated as very low [Table 2] because of moderate heterogeneity and imprecision in the effect estimate, as indicated by the wide confidence interval and limited number of events [Fig. 2].

**Fig. 3:**
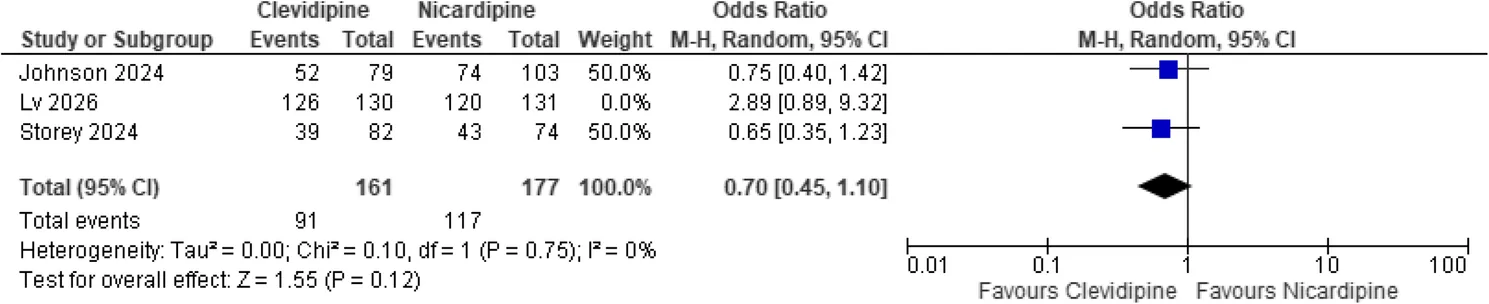
Sensitivity analysis of achievement of target blood pressure reduction

**Fig. 4:**
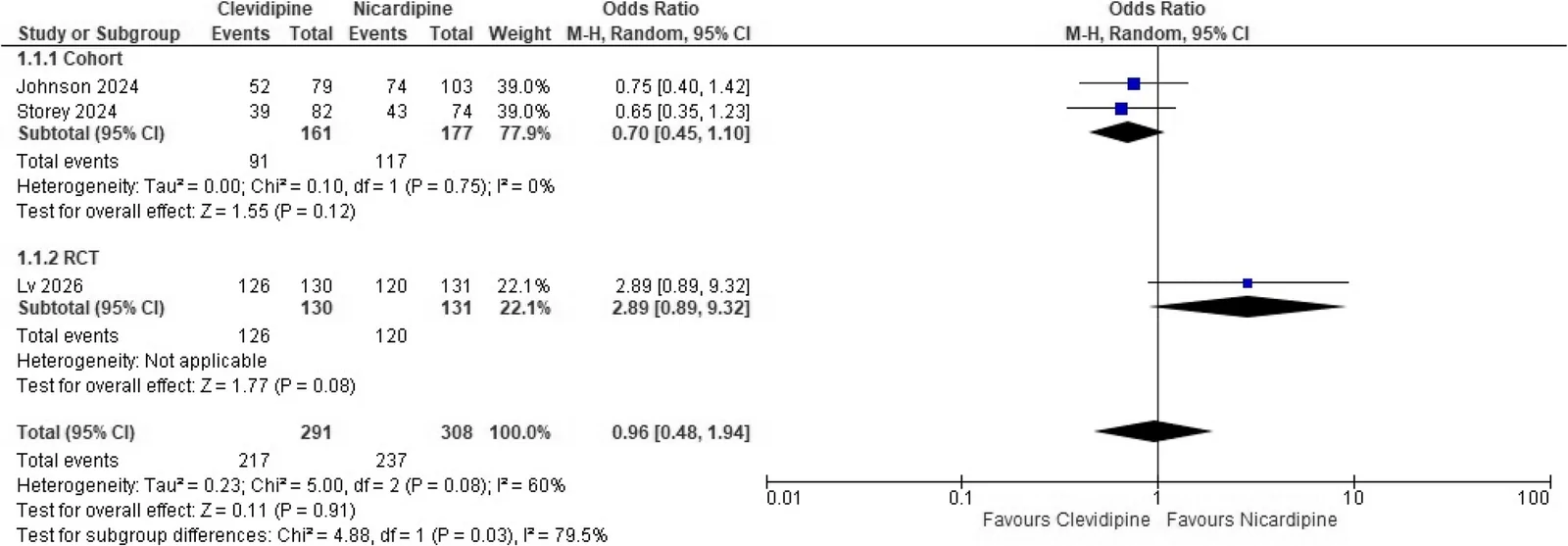
Subgroup analysis of achievement of target blood pressure reduction

A leave-one-out sensitivity analysis was performed by excluding Lv 2026 which eliminated the heterogeneity (OR 0.70, 95% CI 0.45–1.10, *p* = 0.12; I² = 0%) without materially altering the direction of the pooled effect, suggesting that differences in study design, patient population, and treatment protocols contributed to the initial heterogeneity [Fig. 3]. Subgroup analyses according to study design (randomized controlled trial versus observational studies) are presented in [Fig. 4]. These findings should be interpreted cautiously because each subgroup contained only a small number of studies, limiting statistical power.

#### Secondary outcomes

##### Blood pressure over-reduction

**Fig. 5:**
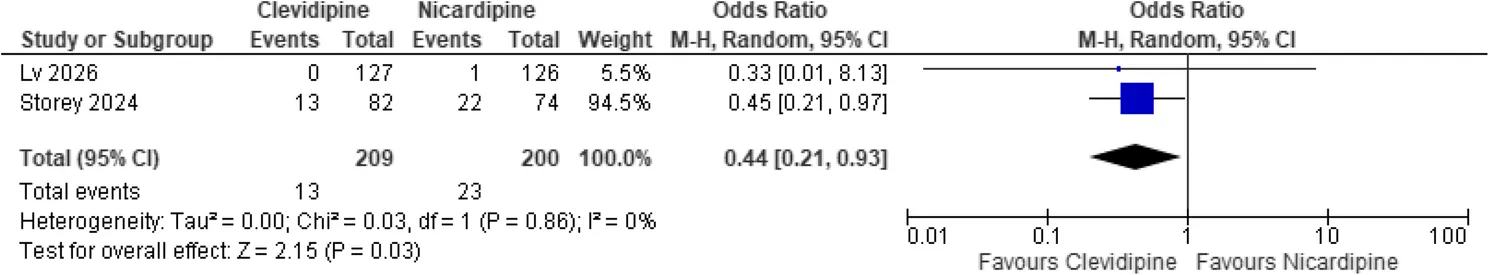
Forest plot of BP Over reduction

Two studies including 409 patients evaluated the risk of blood pressure over-reduction. The pooled analysis showed significantly lower odds of blood pressure over-reduction with clevidipine compared with nicardipine (OR 0.44, 95% CI 0.21–0.93, *p* = 0.03; I² = 0%) [Fig. 5]. The direction of effect was consistent across both studies, with no statistical heterogeneity observed, supporting the consistency of the pooled estimate. The certainty of the evidence was rated as moderate, with no serious concerns identified in any domain [Table 2].

##### Medication volume used

**Fig. 6:**
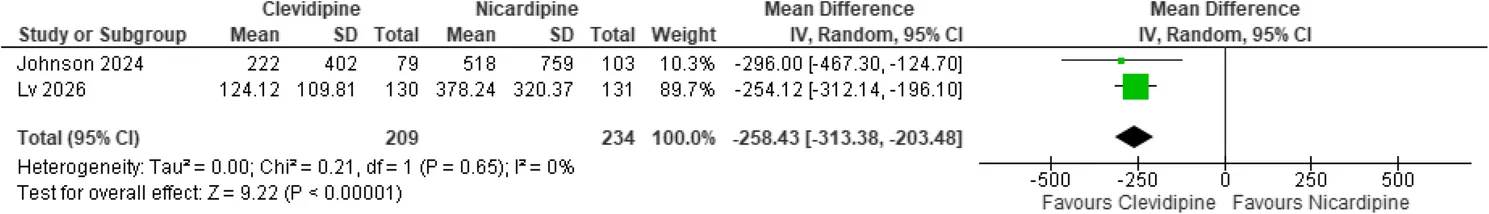
Forest plot of medication volume used

Two studies including 443 patients assessed medication volume. The pooled analysis showed a significantly lower medication volume used with clevidipine compared with nicardipine (MD − 258.43 mL, 95% CI − 313.38 to − 203.48 mL, *p* < 0.00001; I² = 0%) [Fig. 6]. The direction of effect was consistent across both studies, with no statistical heterogeneity observed, supporting the consistency of the pooled estimate. The certainty of the evidence was rated as low because of some concerns regarding risk of bias in the randomized controlled trial, which contributed the greatest weight to the analysis [Table 2].

## Discussion

The current systematic review and meta-analysis reveal that clevidipine and nicardipine were equally efficacious in achieving protocol-defined early blood pressure reduction in patients with hypertensive crises. However, these findings should be interpreted cautiously because the analysis was based on only three studies involving 599 patients, resulting in limited statistical power and increased uncertainty around pooled estimates. Although no statistically significant difference was observed between treatment groups, substantial clinical and methodological variability existed among the included studies. Differences were observed in patient populations (hypertensive emergency versus mixed hypertensive urgency/emergency), blood pressure parameters assessed (systolic blood pressure versus mean arterial pressure), treatment protocols, starting doses, target blood pressure reduction thresholds, timing of outcome assessment, and healthcare settings. These differences may influence treatment response and may not be fully reflected by statistical measures of heterogeneity alone.

Moderate statistical heterogeneity was observed for the primary efficacy outcome. Leave-one-out sensitivity analysis demonstrated that exclusion of the study by Lv et al. eliminated the observed heterogeneity without materially changing the direction of the pooled effect. This suggests that characteristics specific to this study, including differences in study design, patient characteristics, treatment protocols, or outcome assessment, may have contributed to between-study variability. Furthermore, although some analyses demonstrated low statistical heterogeneity, an I² value of 0% should not be interpreted as evidence of complete clinical homogeneity, particularly when based on a small number of studies. Clinical differences across populations and interventions may persist despite limited statistical heterogeneity.

An important finding of this analysis was the lower risk of blood pressure over-reduction observed with clevidipine compared with nicardipine. This finding may suggest more predictable blood pressure control; however, the underlying mechanism cannot be established from this meta-analysis. The pharmacokinetics of clevidipine, such as rapid metabolism by esterases in the bloodstream and tissues, together with a short half-life, may offer a biological explanation for the ease of titration and regulation of blood pressure. Nevertheless, these characteristics should only be regarded as potential modes of explanation rather than evidence of a direct causal relationship [9, 15]. The avoidance of extreme blood pressure lowering remains clinically important, since severe hypotension can negatively impact the perfusion of organs and potentially contribute to adverse outcomes [16].

Clevidipine also required a significantly lower medication volume than nicardipine. Although this finding may reflect differences in infusion requirements, its clinical importance remains uncertain because medication volume is a surrogate pharmacodynamic outcome rather than a patient-centred endpoint. This observation is consistent with previous reports describing effective blood pressure control with clevidipine in acute care settings [17]. Nevertheless, neither medication volume nor early achievement of blood pressure targets should be interpreted as evidence of improved patient-centred outcomes. Although rapid blood pressure control is an important component of hypertensive crisis management, the extent to which these short-term measures translate into reductions in mortality, prevention of end-organ injury, intensive care unit length of stay, hospital length of stay, or other clinically meaningful outcomes remains uncertain [16]. Moreover, several outcomes in this review were derived from only a small number of studies, limiting precision and increasing uncertainty regarding the magnitude of observed effects. Therefore, statistically significant findings should be interpreted within the context of the limited evidence base.

Medication volume reduction should also not be interpreted as evidence of cost-effectiveness. Although clevidipine required a lower volume of administration, the medication itself is generally more expensive than nicardipine in many healthcare systems [13], and none of the included studies formally evaluated economic outcomes. Future studies incorporating cost analyses are needed to determine whether potential advantages in titration efficiency translate into overall healthcare value. In clinical practice, selection of intravenous antihypertensive therapy should consider efficacy, safety, local drug availability, acquisition costs, and institutional treatment protocols.

Although previous investigations have frequently evaluated clevidipine in specific clinical settings, particularly cerebrovascular disease, the comparative evidence between clevidipine and nicardipine remains limited. Earlier analyses have reported similar efficacy between the two agents while highlighting differences in pharmacodynamic profiles [8]. In contrast, some retrospective studies have shown no significant difference in initial blood pressure control across intravenous antihypertensive agents [5], suggesting that clinical context and patient characteristics may influence treatment response.

Safety outcomes reported in prior literature have been variable. For example, Rosenfeldt et al. described a higher incidence of hypotension with clevidipine in a small cohort; however, these findings were limited by sample size and study design [18]. Other studies have reported no significant differences in adverse events between clevidipine and comparator agents, supporting the overall safety of both treatments [8, 9]. The present analysis contributes to this literature by suggesting a lower risk of blood pressure over-reduction with clevidipine. However, the clinical importance of this finding remains uncertain because absolute event rates were low, and the available studies were not designed or powered to determine whether differences in blood pressure over-reduction translate into improvements in mortality, organ dysfunction, or healthcare utilization. In addition, a reliable number needed to treat (NNT) could not be calculated because only two studies contributed to this outcome, and consistent absolute risk differences were not available across the included studies. Relative treatment effects should therefore be interpreted cautiously in the absence of demonstrated improvements in patient-important outcomes.

From a clinical perspective, both clevidipine and nicardipine appear to be effective intravenous antihypertensive agents for achieving early blood pressure control. Clevidipine may offer practical advantages due to its rapid onset of action, short duration of effect, and ease of titration; however, these potential benefits should be interpreted in the context of the limited comparative evidence and very low certainty of evidence for the primary efficacy outcome. Given the small number of available studies and methodological limitations, the findings should be considered hypothesis-generating rather than definitive. 

### Limitations

Several limitations should be considered when interpreting the findings of this meta-analysis. Only three studies involving 599 patients were included, resulting in limited statistical power and increased uncertainty around the pooled estimates. Second, the evidence base included one randomized controlled trial and two observational studies. Because of the limited number of comparative studies, randomized and observational evidence were synthesized to provide an overall estimate; however, this approach introduces uncertainty because observational studies remain susceptible to residual confounding and selection bias. Therefore, the findings should be interpreted as a synthesis of the currently available evidence rather than definitive comparative effectiveness estimates. Furthermore, differences in study populations, treatment protocols, blood pressure targets, outcome definitions, and healthcare settings could have contributed to clinical heterogeneity that could not be fully captured by statistical measures such as the I² statistic. Although all included studies evaluated early achievement of protocol-defined blood pressure reduction following initiation of intravenous antihypertensive therapy, differences existed in the blood pressure metric assessed (SBP versus MAP), target reduction thresholds, and timing of assessment. These variations may have contributed to inconsistency in the pooled estimates. Formal assessment of publication bias was not performed because fewer than ten studies were available for each outcome, consistent with recommended methodological guidance. Finally, only English-language, peer-reviewed studies were included, while grey literature, conference abstracts, and unpublished studies were excluded, which may have introduced publication and language bias. 

### Strengths

Despite these limitations, this meta-analysis provides a comprehensive synthesis of the available comparative evidence between clevidipine and nicardipine in hypertensive crises. Strengths of this review include protocol registration, a comprehensive literature search, standardized risk-of-bias assessment, sensitivity analyses, and application of the GRADE framework. However, certainty of evidence varied across outcomes, with very low certainty for the primary efficacy outcome, emphasizing the need for cautious interpretation and highlighting the requirement for stronger comparative evidence.

Future research should focus on adequately powered randomized controlled trials using standardized blood pressure targets, consistent outcome definitions, and uniform assessment time points. Additional studies evaluating long-term clinical outcomes, safety, and cost-effectiveness are required to better determine the comparative role of clevidipine and nicardipine in hypertensive crises.

## Conclusion

Current evidence suggests that clevidipine and nicardipine provide comparable efficacy in achieving target blood pressure reduction among patients with hypertensive crises. Although clevidipine was associated with lower odds of blood pressure over-reduction and lower medication volume, whether these differences translate into improved patient-centred outcomes remains uncertain. Given the limited evidence base and the very low certainty of evidence for the primary outcome, these findings should be considered hypothesis-generating rather than definitive. Larger, well-designed randomized controlled trials evaluating standardized outcomes and patient-centred endpoints are required before firm conclusions regarding comparative effectiveness, safety, or treatment preference can be established. 

## Supplementary Information

Supplementary Material 1.

Supplementary Material 2.

Supplementary Material 3.

## Data Availability

All data analyzed in this study are derived from previously published articles, which are cited in the reference list. The extracted dataset supporting the findings of this study is included within the article and its supplementary materials.
