# Supplementary material for: Clevidipine vs. nicardipine in hypertensive crisis: a systematic review and meta-analysis
Source: BMC Cardiovasc Disord. 2026 Jul 24;26:942. doi: 10.1186/s12872-026-06345-z (PMC13681746; doi:10.1186/s12872-026-06345-z)
Supplement: Supplementary file 1 [file 12872_2026_6345_MOESM1_ESM.docx]

| **Primary Outcomes** | **Outcome** | **Definition** |
| --- | --- | --- |
| 1 | Achievement of target blood pressure reduction | Achievement of the predefined target blood pressure reduction following initiation of intravenous antihypertensive therapy, as defined by each included study. The blood pressure metric (e.g., SBP or MAP), target reduction, and timing of assessment was extracted and analyzed as originally reported. \| |
| 2 | Blood Pressure Over-Reduction | Incidence of excessive blood pressure lowering below predefined safety thresholds, as defined by the original studies. |
| **Secondary Outcomes** | **Outcome** | **Definitions** |
| 1 | Medication Volume Used | Total volume or dose of intravenous antihypertensive medication administered during the treatment period. |
| 2 | Intensive Care Unit Length of Stay (ICU LOS) | Duration of ICU admission, measured in hours or days, following initiation of antihypertensive therapy. |
| 3 | Time to Blood Pressure Reduction | Time elapsed from initiation of intravenous antihypertensive therapy to achievement of target or clinically acceptable blood pressure, as defined by each study. |

**Supplementary Table S1*:*** Definition of Outcomes

| **Author,**  **Year** | **Country** | **Route of Administration** | **Type of Study** | **Intervention** | **Control** | **Reason Excluded** |
| --- | --- | --- | --- | --- | --- | --- |
|  |  |  |  |  |  |  |
| Schalk 2022 | USA | IV | Cohort | Clevidipine | Nicardipine | Stroke Population |
| Saldana 2021 | USA | IV | Cohort | Clevidipine | Nicardipine | Stroke Population |
| Allison 2019 | USA | IV | Cohort | Clevidipine | Nicardipine | Stroke Population |
| Rosenfeldt 2019 | USA | IV | Cohort | Clevidipine | Nicardipine | Stroke Population |
| Finger 2017 | USA | IV | Cohort | Clevidipine | Nicardipine | Stroke Population |
| Armstrong 2025 | USA | IV | Cohort | Clevidipine | Nicardipine | Stroke. ACS, Dissection |
| Borrell Vega 2020 | USA | IV | Cohort | Clevidipine | Nicardipine | Stroke |
| Rodriguez 2022 | USA | IV | Cohort | Clevidipine | Nicardipine | Pre-print |
| Nicardipine 2021 | USA | IV | Cohort | Clevidipine | Nicardipine | Stroke |
|  | | | | | | |

**Supplementary Table S2*:*** Excluded Studies
