# Supplementary material for: Clevidipine vs. nicardipine in hypertensive crisis: a systematic review and meta-analysis
Source: BMC Cardiovasc Disord. 2026 Jul 24;26:942. doi: 10.1186/s12872-026-06345-z (PMC13681746; doi:10.1186/s12872-026-06345-z)
Supplement: Supplementary file 2 [file 12872_2026_6345_MOESM2_ESM.docx]

Risk of Bias Assessment:

Lv et. all (2026) Using ROB 2.0:

***Risk of Bias Assessment for Storey (2024) and Johnson (2024) using Newcastle-Ottawa Scale (NOS):***

| Study ID | SELECTION | | | | COMPARIBILITY | | OUTCOMES | | | TOTAL |
| --- | --- | --- | --- | --- | --- | --- | --- | --- | --- | --- |
|  | S1 | S2 | S3 | S4 | C1 | C2 | O1 | O2 | O3 |  |
| Storey et al.2024 | * | * | * | _ | * | * | * | * | * | 8 |
| Johnson et al. 2024 | * | * | * | _ | * | * | * | * | * | 8 |

Newcastle–Ottawa Scale (NOS) Quality Assessment

Storey et al.2024

| Selection | Item | Stars | Rationale |
| --- | --- | --- | --- |
| Selection | Representativeness of exposed cohort | 1 | Cohort reflects typical patients presenting with hypertensive crisis in a real-world hospital setting. |
| Selection | Selection of non-exposed cohort | 1 | Historical comparison group from the same hospital, treated with the alternative drug. |
| Selection | Ascertainment of exposure | 1 | Exposure (nicardipine or clevidipine) determined from electronic health records and infusion logs. |
| Selection | Outcome not present at start | 0 | outcomes occur after treatment, not present at baseline. |
| Comparability | Controlled for primary confounder | 1 | Baseline characteristics were compared using various test. |
| Comparability | Controlled for additional factors | 1 | Present |
| Outcome | Assessment of outcome | 1 | Outcomes like MAP reduction and SBP control clearly defined and assessed from patient records. |
| Outcome | Follow-up long enough | 1 | Acute BP outcomes measured at 1–6 hours; sufficient for hypertensive crisis evaluation. |
| Outcome | Adequacy of follow-up | 1 | No patients were lost from the retrospective database used for analysis |

Total Stars =8/9

Johnson et al. 2024

| Selection | Item | Stars | Rationale |
| --- | --- | --- | --- |
| Selection | Representativeness of exposed cohort | 1 | ICU patients from multiple hospital types, typical for hypertensive crisis. |
| Selection | Selection of non-exposed cohort | 1 | Comparison group from same hospitals and period, fair and comparable. |
| Selection | Ascertainment of exposure | 1 | Drug assignment obtained from reliable EMR data |
| Selection | Outcome not present at start | 0 | outcomes occur after treatment, not present at baseline. |
| Comparability | Controlled for primary confounder | 1 | Baseline characteristics were compared using various tests. |
| Comparability | Controlled for additional factors | 1 | Present |
| Outcome | Assessment of outcome | 1 | BP measurements obtained from EMR , outcome clearly defined. |
| Outcome | Follow-up long enough | 1 | Time to goal BP occurs within minutes to hours ,ICU stay recorded. |
| Outcome | Adequacy of follow-up | 1 | Complete outcome data available from EMR , no significant loss to follow-up. |

Total Stars =8/9
