# Supplementary material for: Clevidipine vs. nicardipine in hypertensive crisis: a systematic review and meta-analysis
Source: BMC Cardiovasc Disord. 2026 Jul 24;26:942. doi: 10.1186/s12872-026-06345-z (PMC13681746; doi:10.1186/s12872-026-06345-z)
Supplement: Supplementary file 3 [file 12872_2026_6345_MOESM3_ESM.docx]

**Funnel Plot for Outcomes:**

Figure 1: 15-25% SBP Reduction within 1 hour

Figure 2: Medication Volume

Figure 3: BP Over Reduction
